# Recent Advances of Enzymes in the Food Industry

**DOI:** 10.3390/foods12244506

**Published:** 2023-12-17

**Authors:** Wenhua Yang, Fuping Lu, Yihan Liu

**Affiliations:** Key Laboratory of Industrial Fermentation Microbiology, Ministry of Education, Tianjin Key Laboratory of Industrial Microbiology, College of Biotechnology, Tianjin University of Science and Technology, Tianjin 300457, China; yangwenhua@tust.edu.cn

## 1. Introduction

Enzymes used in the food industry are obtained from plants, animals, or microorganisms. Food enzymes not only aid in the breakdown and digestion of food but also are used to speed up food processing and improve the quality of food products [1]. Food enzymes, including amylase, protease, lipase, oxidoreductase, and isomerase, are important food processing aids in the food industry. They are used in a variety of fields, such as starch processing, dairy products, bakery products, spices, fermented products, food additives, wine, and beverages. The size of the global food enzymes market reached USD 2058 million in 2020 and is expected to reach USD 4194 million in 2032, at a revenue compound annual growth rate (CAGR) of 6.1% [2]. The market for food enzymes maintains a continuous upward trend and has great potential for future growth.

Food enzymes play an increasingly important role in the food industry. Discovering new food enzymes and modifying them are key to optimizing food processing and improving product quality. In the past, researchers have obtained food enzymes with specific functions by screening microorganisms and through purification and identification of the proteins, which is inefficient and needs to be explored further [3]. In addition, it is important to modify the discovered food enzymes to improve or optimize their properties and catalytic efficiency. Although microbial fermentation has become the mainstream method for the production and application of food enzymes, further increasing the yield while also reducing the cost is still an emerging problem. Furthermore, the effective application of enzymes in the food industry needs to be further explored. The research, production, and application of food enzymes still face many challenges. Thus, it is necessary to explore and solve these issues in depth to achieve greater breakthroughs in the research and application of food enzymes.

## 2. Mining and Modification of Food Enzymes

Novel food enzymes occupy an important position in the food industry and are expected to overcome the deficiencies of existing enzymes. In the past, novel functional enzymes were often obtained through screening microorganisms and purifying them from microbial total proteins. With the discovery of next-generation sequencing technologies, the discovery efficiency of novel food enzymes has been greatly improved [4]. Genomics and bioinformatics approaches enable screening and cloning of the coding sequences of enzymes from nature with unknown functions and allow us to predict their function. Metabolomics is the study of changes in metabolite fractions of organisms under different physiological conditions to discover metabolites related to enzyme action and, thus, to find novel enzymes. Moreover, proteomics searches for new enzyme species by analyzing the structure and function of various enzymes. Furthermore, structural biology is based on the three-dimensional atomic structure of enzymes, combined with computer simulation techniques to predict and verify enzyme functions and to design programs to modify enzymes. Each of these new food enzyme mining technologies has unique characteristics, and the most appropriate technology can be selected according to the actual needs. Meanwhile, the continuous development and integration of these technologies will provide more possibilities for food enzyme mining.

In addition to mining novel food enzymes, modification of food enzymes can also improve enzyme performance, such as enhancing enzyme stability, reducing antigenicity, and increasing catalytic efficiency [5]. These improved properties can make it more well-suited for the needs of food processing and storage. Food enzymes can be modified using various methods. The molecular structure of an enzyme can be chemically altered, for example, the hydrophilicity and stability of the enzyme can be increased by adding amino acids, sugars, and other groups. The properties of enzymes can be altered through genetic engineering, including targeted mutagenesis, combinatorial active-site saturation test, iterative saturation mutagenesis, and structural domain deletions or substitutions using artificial intelligence designs based on their structures. The stability and reusability of enzymes can be improved by immobilizing enzymes on certain carriers through embedding or immobilization techniques. For instance, immobilizing the enzyme on ion exchange resins or nanoparticles can increase the adsorption capacity and hydrolysis efficiency of the enzyme. Of course, combining multiple modification methods can improve several performance indicators of enzymes at the same time. Additionally, the modification of food enzymes needs to follow food safety and regulatory requirements without introducing any substances or genes that are harmful to health.

## 3. Production and Application of Food Enzymes

Increasing the production of food enzymes is of great significance to the development of the food industry, which can reduce production costs, improve the quality of products, simplify processing technology, meet market demand, and enhance the competitiveness of enterprises. The high-level production of food enzymes is a comprehensive process that requires multifaceted approaches, and a variety of technologies have been developed and applied to increase enzyme yields [6]. Genetic methods, such as the use of host cells with high expression levels of secreted proteins, constructing suitable genetic engineering vectors, codon optimization for coding nucleic acids, screening of host cells containing multiple copies of target genes, and selection of strains with high enzyme production through mutagenesis and in vivo recombination techniques, have been explored for improving the production of enzymes. In addition, optimizing the culture medium and the culture conditions, adding inducers, controlling the concentration of deterrents, adding surfactants, and adding enzyme-producing enhancers can also improve the production of food enzymes. However, the application of these biotechnologies needs to be selected and optimized according to specific production conditions and actual needs.

Food enzymes have a variety of functions that result in their extensive use in the food industry [7]. First, the most important function of food enzymes is that they can break down the macromolecular compounds in food into small molecular compounds that can be more easily absorbed and utilized by the human body, e.g., proteases can hydrolyze proteins into low molecular peptides. Secondly, food enzymes can oxidize or reduce certain compounds in food into compounds with different chemical properties, e.g., catalase can oxidize hydrogen peroxide to oxygen, thereby extending the shelf life of food. Thirdly, food enzymes have an isomerization function that allows them to change the structure of compounds to give them different properties. For example, esterases can hydrolyze fats and oils, converting them into fatty acids and glycerol, while fatty acids can be re-esterified into fats and oils. Furthermore, food enzymes can polymerize low-molecular-weight compounds into high-molecular-weight compounds. For instance, transglutaminase can polymerize proteins in muscle fibers to improve the tenderness and texture of meat products.

## 4. Innovation and Development of Food Enzymes

Technological innovation plays a particularly important role in upgrading and transforming the food enzyme industry [8]. Enzyme sensors developed through biosensor technology can detect harmful substances and nutrients in food, thus improving the safety and nutritional value of food. The de novo design of enzymes makes it possible to create non-natural enzymes, and this approach breaks the limitations of traditional enzyme discovery and provides a completely new method for enzyme modification. In addition, the use of nanotechnology and microencapsulation technology to make enzymes into nanoscale microspheres or microcapsules during food processing not only improves the stability and catalytic properties of enzymes but also enhances the storage time and application range of enzymes. With the development of synthetic biology, some researchers use cell factories to produce food additives such as colors, sweeteners, and preservatives. The development of these new technologies provides broader prospects for the application of food enzymes. At the same time, they also provide further possibilities for developing the food industry.

The developing trend of food enzymes is influenced by several factors, such as market demand, technological advancements, and industry regulations. Food enzymes are facing higher challenges as consumers demand more nutritious, healthy, and safe food. Therefore, the development of high-activity and high-quality composite enzymes has become one of the main directions of food enzyme research. In addition, the existence of such a wide variety of food ingredients places higher demands on the catalytic ability, catalytic conditions, and tolerance of enzymes. Hence, customized enzymes for different food ingredients and nutritional requirements will likely become a trend. Moreover, enzymes may have residual problems in some cases which affect the safety and hygiene quality of food products. Thus, an effective quality control system needs to be established during food processing to ensure that enzyme residues comply with relevant national standards. With the continuous progress of science and technology, it is believed that the application of food enzymes will be more diversified in the future, thus bringing new opportunities and challenges to the development of the food industry.
